# The Mechanism Switching the Osteoclast From Short to Long Duration Bone Resorption

**DOI:** 10.3389/fcell.2021.644503

**Published:** 2021-03-30

**Authors:** Jean-Marie Delaisse, Kent Søe, Thomas Levin Andersen, Aleksandra Maria Rojek, Niels Marcussen

**Affiliations:** ^1^Clinical Cell Biology, Department of Pathology, Odense University Hospital, Odense, Denmark; ^2^Clinical Cell Biology, Pathology Research Unit, Department of Clinical Research, University of Southern Denmark, Odense, Denmark; ^3^Department of Molecular Medicine, University of Southern Denmark, Odense, Denmark; ^4^Department of Forensic Medicine, Aarhus University, Aarhus, Denmark

**Keywords:** osteoporosis, ruffled border, resorption trenches, cathepsin K, collagen, sealing zone, osteoclast

## Abstract

The current models of osteoclastic bone resorption focus on immobile osteoclasts sitting on the bone surface and drilling a pit into the bone matrix. It recently appeared that many osteoclasts also enlarge their pit by moving across the bone surface while resorbing. Drilling a pit thus represents only the start of a resorption event of much larger amplitude. This prolonged resorption activity significantly contributes to pathological bone destruction, but the mechanism whereby the osteoclast engages in this process does not have an answer within the standard bone resorption models. Herein, we review observations that lead to envision how prolonged resorption is possible through simultaneous resorption and migration. According to the standard pit model, the “sealing zone” which surrounds the ruffled border (i.e., the actual resorption apparatus), “anchors” the ruffled border against the bone surface to be resorbed. Herein, we highlight that continuation of resorption demands that the sealing zone “glides” inside the cavity. Thereby, the sealing zone emerges as the structure responsible for orienting and displacing the ruffled border, e.g., directing resorption against the cavity wall. Importantly, sealing zone displacement stringently requires thorough collagen removal from the cavity wall - which renders strong cathepsin K collagenolysis indispensable for engagement of osteoclasts in cavity-enlargement. Furthermore, the sealing zone is associated with generation of new ruffled border at the leading edge, thereby allowing the ruffled border to move ahead. The sealing zone and ruffled border displacements are coordinated with the migration of the cell body, shown to be under control of lamellipodia at the leading edge and of the release of resorption products at the rear. We propose that bone resorption demands more attention to osteoclastic models integrating resorption and migration activities into just one cell phenotype.

## 1. An Osteoclastic Resorption Event Often Goes Beyond the Formation of a Pit

**FIGURE 1:**
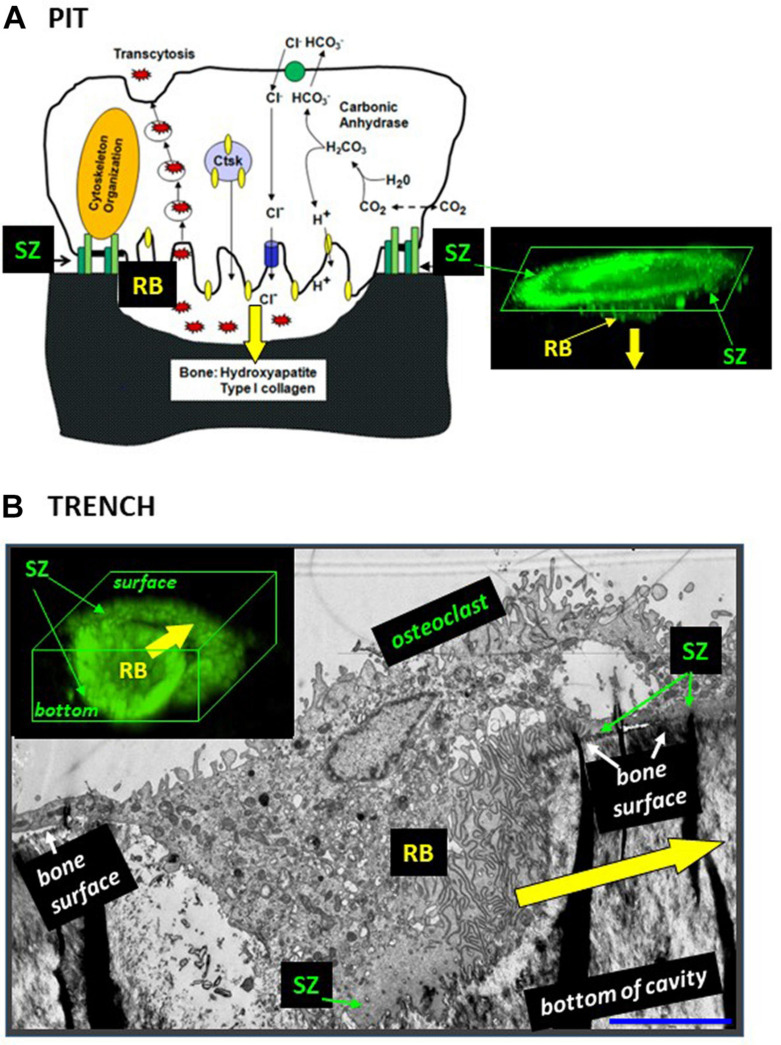
The configuration of the ruffled border and the SZ is different in osteoclasts making pits **(A)** and trenches **(B)**. **(A)** Pit mode. Classical model of the bone resorption mechanism [reproduced from Feng and Teitelbaum (2013) with permission] and confocal microscopy image showing the corresponding 3D configuration of the SZ and the ruffled border (RB) [reproduced from Søe and Delaissé (2017) with permission; SZ and ruffled border were visualized by staining actin with phalloidin]. Note the planar SZ on the bone surface. It surrounds the ruffled border and consequently the ruffled border is directed against the bone surface, so that resorption occurs perpendicularly to the bone surface (yellow arrow). **(B)** Trench mode. Osteoclasts were generated from blood donors and cultured on bone slices. An osteoclast was sectioned parallel to its resorption/migration axis (identified beforehand through light microscopy), processed for electron microscopy (Kristensen et al., 2013), and a picture was taken (unpublished). The corresponding 3D configuration of the SZ and the ruffled border (RB) is shown on a confocal picture [reproduced from Søe and Delaissé (2017) with permission; stainings as in panel **(A)**]. Note that the front side of the SZ is on the bone surface and the rear side of the SZ in the cavity. Consequently, the ruffled border is rotated compared with the pit mode and resorbs parallel to the bone surface (yellow arrow). Scale bar in panel **(B)**: 5 μm.

Bone resorption is necessary to allow bone modeling and remodeling, and may become deleterious in pathological situations. The cell performing bone resorption is the multinucleated osteoclast. The main components of the resorption machinery have been identified (Väänänen et al., 2000; Coxon and Taylor, 2008; Feng and Teitelbaum, 2013; Ng et al., 2019) and are usually pictured as in Figure 1A (shown here for the main subcellular components – not for the molecular details). (i) The ruffled border is a zone of the plasma membrane facing the bone surface and is specialized in secretion of resorption agents and uptake of resorption products - much like the boring head of a tunneling machine integrating destruction and evacuation of debris. (ii) The sealing zone (SZ) surrounds the ruffled border and delineates the area of bone surface to be resorbed. Together with the ruffled border it delimits the actual “subosteoclastic resorption compartment.” The SZ is commonly believed to prevent the diffusion of resorption agents to the surrounding bone surface and to anchor the bone resorption compartment to the bone surface. (iii) An intricate network of vesicles trafficking through the cell deliver resorption agents to the resorption compartment and transport resorption products from the ruffled border to the basolateral domain. (iv) A complex cytoskeletal organization is involved in this intracellular trafficking and connected to the SZ. Of note, in this setup the SZ is positioned on the native bone surface as a planar ring surrounding the ruffled border (Figure 1A). In this way, the ruffled border invades the bone matrix perpendicularly to the bone surface and generates a pit (Figure 1A). According to this model, the resorption event remains stationary on the bone surface for its whole duration (typically 2-24 h in bone resorption assays) (Søe and Delaissé, 2017). This model became the standard reference of most studies. We refer to it as the pit resorption mode (Søe and Delaissé, 2010, 2017; Søe et al., 2013; Merrild et al., 2015).

**FIGURE 2:**
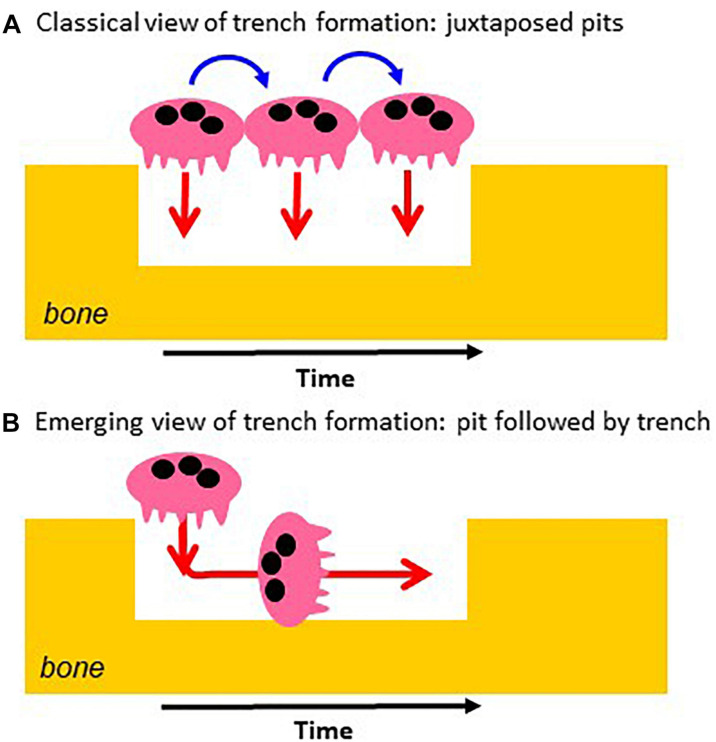
Classical vs. novel view of the mechanism of trench formation. **(A)** The classical view of trench formation is based on the traditional pit model and ascribed to successive pit formations (red arrows). They are separated by cell migration/stretching episodes (blue arrows). **(B)** The novel view is directly based on the course of events revealed by time lapse: the osteoclast starts making a pit and then enlarges it laterally. The change of orientation of the resorption axis results from the re-orientation of the ruffled border (see Figure 1).

**FIGURE 3:**
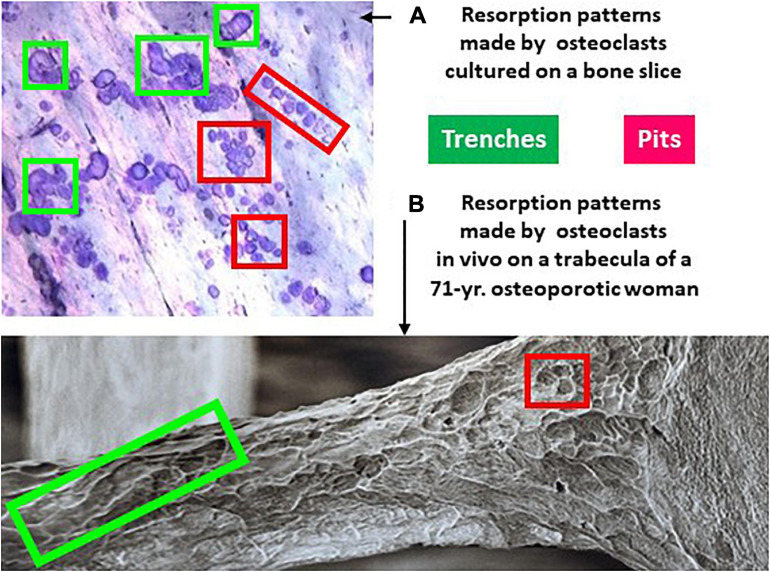
Resorption patterns of osteoclasts in culture **(A)** and *in vivo* **(B)** show both pits and trenches. **(A)** Picture of the surface of a bone slice exposed to the resorptive activity of osteoclasts generated from blood donors (unpublished illustration). The slices were stained with toluidine blue as explained in Søe and Delaissé, 2010. **(B)** Trabecula of the third lumbar vertebra of a 71-year old osteoporotic woman, processed for scanning electron microscopy (reproduced from the British Bone Research Society with kind permission of Tim Arnett, University College London). Both **(A**,**B)** show two types of resorption cavities: trenches representing continuous resorption events parallel to the bone surface (green frames), and pits representing resorption events perpendicular to the bone surface. Pits are often seen in groups - which reflects repeated resorption events separated by migration episodes.

It follows from this standard view that extension of the resorption activity to the bone surface and enlargement of the initial pit were proposed to occur by repeated pit formations, separated by migration/stretching episodes (Lakkakorpi and Vaananen, 1991; Saltel et al., 2004; Novack and Faccio, 2011; Vives et al., 2011; Rumpler et al., 2013; Georgess et al., 2014; Figure 2A). Thus the elongated excavations on bone surfaces, called herein trenches (Figure 3), were ascribed to a series of confluent pits (Figure 2A). Clusters or trails of pits generated by osteoclasts cultured on bone slices were considered to support the existence of alternations between pit formation and migration episodes (Figure 3). Recent time lapse observations of these osteoclasts directly confirmed this hypothesis (Søe and Delaissé, 2017). However, time lapse revealed as well that many osteoclasts do not stop resorbing after the generation of a pit, but instead enlarge it laterally thereby making a trench (Søe and Delaissé, 2017). These time lapse observations also stressed that when the osteoclasts do so, they do not disassemble the extracellular resorption compartment as commonly believed (Lakkakorpi and Vaananen, 1991; Saltel et al., 2004; Georgess et al., 2014), but instead displace it continuously parallel to the bone surface (Søe and Delaissé, 2017). Thus one should be aware that pit formation is for many osteoclasts only the start of a resorption event of much larger amplitude [typically days in bone resorption assays (Søe and Delaissé, 2017)] (Figure 2B). We call this process the trench resorption mode (Søe and Delaissé, 2010, 2017; Søe et al., 2013; Merrild et al., 2015; Borggaard et al., 2020).

This newly discovered trench resorption mode deserves much attention. First, trenches represent typically more than 50% of the resorption events achieved on bone slices by osteoclasts generated from blood donors (Søe and Delaissé, 2017). Thus more than 50% of the osteoclasts are enlarging their original pit in this bone resorption assay. Second, the trench resorption mode shows higher resorption performances in terms of duration, speed, and depth, compared with the pit resorption mode, and is thus more aggressive (Merrild et al., 2015; Søe and Delaissé, 2017; Table 1). Of note, less than 15% of the osteoclasts that started to make a trench stop resorbing within a 72 h culture period (Søe and Delaissé, 2017; Borggaard et al., 2020). Third, the trench mode appears to have clinical relevance (Table 1). Glucocorticoids were found to increase the prevalence of trenches generated in the *in vitro* resorption assays (Søe and Delaissé, 2010), and significantly more trenches were produced by osteoclasts generated from aged blood donors (Møller et al., 2020c). More trenches were also produced by osteoclasts generated from males compared to females (Merrild et al., 2015). In contrast, pharmacological agents like bisphosphonates decrease the prevalence of trenches (Møller et al., 2020a) and cathepsin K inhibitors completely abrogate them (Søe et al., 2013; Merrild et al., 2015; Panwar et al., 2016, 2017; Borggaard et al., 2020). Furthermore, pit and trenches appear to differently affect bone strength (Vanderoost et al., 2013). Scanning electron microscopy pictures of bone surfaces of osteoporotic patients, which are regularly used to highlight extended erosion, actually show a high prevalence of trenches (Figure 3B). Thus trenches are also generated *in vivo* and their prevalence in osteoporosis seems to have been mostly overlooked. Overall, it thus seems that the trench mode, i.e., cavity enlargement, is an important contributor to pathological bone resorption.

**TABLE 1:** Overview of differences between the pit and trench resorption mode.

	Pit mode* Standard model	⟶	*Trench mode *Mostly disregarded*	References
	
Basic characteristics	• Resorbs without moving	• Resorbs while moving		Søe and Delaissé, 2017
	• Drills perpendicularly to the bone surface	• Erodes parallel to the bone surface *(enlarging laterally the initial pit)*		Søe and Delaissé, 2017
	• Short duration (hours)	• Long duration (days)		Søe and Delaissé, 2017
	• Slower (50 μm^2^/h)	• Faster (100 μm^2^/h)		Søe and Delaissé, 2017
	• Shallower (8 μm)	• Deeper (12 μm)		Merrild et al., 2015
Mechanistic features	• Collagenolysis rate slower than demineralization rate • Lower active cathepsin K level	• Balanced rate of collagenolysis and demineralization • Higher active cathepsin K level		Søe et al., 2013 and section 3.2 Borggaard et al., 2020 and section 3.3
	• Sealing zone (SZ) on bone surface (*planar – believed to anchor the ruffled border*)	• Sealing zone (SZ) partially in cavity (*(folded – believed to orient and transport the ruffled border)*		Søe and Delaissé, 2017 and section 2
	• No detection of phagocytic features	• Phagocytic features		Borggaard et al., 2020 and section 6
	• No significant relation with number of nuclei per osteoclast	• Increases with number of nuclei per osteoclast		Møller et al., 2020c
Clinical significance		• Increased contribution: with glucocorticoids with age in males		Søe and Delaissé, 2010 Møller et al., 2020c Merrild et al., 2015
		• Abolished by cathepsin K inhibitors		Søe et al., 2013; Merrild et al., 2015; Panwar et al., 2016, 2017; Borggaard et al., 2020
		• Decreased by bisphosphonates		Møller et al., 2020a
	• Appears to favor coupling with bone formation			Delaisse et al., 2020

See section 1 for the definition of pit and trench modes. *Note that in a typical *in vitro* experiment, (performed with osteoclasts generated from CD14-positive monocytes from blood donors) 90% of the trench formation events start as a pit which is thereafter enlarged laterally, whereas half of the resorption events do not go beyond the formation of a pit (Søe and Delaissé, 2017).

It is obviously important to know the mechanism driving resorption beyond the formation of a pit. Knowledge of this mechanism is expected to answer a number of intriguing questions of relevance for the clinic. What determines the duration of a resorption event and the size of the excavation? What controls the orientation of the resorption axis in other directions than perpendicular to the bone surface as in the pit mode? How is simultaneous resorption and displacement of the SZ possible, since they are classically considered mutually exclusive (Lakkakorpi and Vaananen, 1991; Takahashi et al., 2007; Novack and Faccio, 2011; Georgess et al., 2014)? Why is resorption in the trench mode more powerful? We review herein both published and unpublished observations that may help understanding the mechanistic peculiarities of the trench mode vs. the pit mode and thereby help comprehending the resorption power beyond what is classically investigated. They let us proposing a previously underestimated regulatory role of the collagenolysis rate vs. demineralization rate, as it determines whether the SZ can move into the excavation - thereby re-orienting the ruffled border and the resorption axis for cavity enlargement. This leads to envision a bone resorption model that takes into account the full resorption program. Overall, the present analysis invites to improve our view of the bone resorbing osteoclast by taking into account combined resorption and migration.

## 2. A Key Event for Enlargement of Resorption Cavities: The SZ “Stepping” in the Cavity and Reorienting the Ruffled Border

Osteoclasts initiating bone resorption show SZs positioned on the native bone surface as a planar ring surrounding the ruffled border (Figure 1A). As mentioned above, in this way, the ruffled border invades the bone matrix perpendicularly to the bone surface and generates a pit. This invasion is unlikely to be able to proceed beyond a certain depth by simple extension of the ruffled border. Interestingly in this regard, osteoclasts forming a trench have only the leading edge of the SZ positioned on the native bone surface, whereas the rear part of the SZ is lining the freshly resorbed walls of the cavity (making the SZ appearing as a crescent when viewed from above: see e.g., Mulari et al., 2003; Rumpler et al., 2013; Merrild et al., 2015; Figure 1B). Important to note, this means that the transition from the pit to the trench mode goes along with the displacement of the rear part of the SZ into the excavation. This displacement re-orients the penetration angle of the ruffled border into the bone matrix: it becomes directed against one of the walls of the excavation so that the cavity enlarges in this direction – which is parallel to the bone surface (Figures 1, 2B). Further enlargement in a given direction, thus generating a trench, goes along with the continuous displacement of the rear part of the SZ along the walls of the trench, and of the leading edge of the SZ on the bone surface (Figure 1B).

## 3. Thorough Collagen Degradation is Mandatory for Displacement of the SZ in the Resorption Cavity

### 3.1. Collagen Prevents SZ Formation Required for Initiation of Bone Resorption

Common experience is that osteoclasts seeded on bone slices (obtained by sawing and therefore exposing mineralized bone matrix) readily generate a SZ and start resorbing. However, it is also well established that when osteoclasts are exposed to collagen surfaces, they do not show a SZ and do not start resorbing (Saltel et al., 2004; Takahashi et al., 2007). So, osteoclasts are unable to resorb non-mineralized bone in rickets (Nordahl et al., 2000), and osteoclasts cannot resorb native bone surfaces unless the superficial layer of collagenous matrix has been removed (Chambers et al., 1985). Actually, osteoclasts on collagen present a set of distinct phenotypic characteristics (Sato et al., 1998; Saltel et al., 2004; Takahashi et al., 2007). These have been described as a mesenchymal migratory phenotype (Søe and Delaissé, 2010, 2017; Søe et al., 2013), because the response of the osteoclast to collagen vs. bone is reminiscent of epithelial-mesenchymal transitions (Kalluri et al., 2009). One of the characteristics of osteoclasts on collagen is that podosomes are either isolated, or in clusters, or in belts (Saltel et al., 2004; Takahashi et al., 2007). These podosomes are mechanosensing the rigidity of the surface they are in contact with (van den Dries et al., 2019). If the collagen becomes mineralized, the podosomes sense greater rigidity and recognize that the cell is dealing with bone, thereby “authorizing” the activation of the degradation machinery (Saltel et al., 2004). Thus podosomes appear to contribute in making sure that osteoclasts degrade only bone (and only when presenting a “collagen-free” surface) and no other tissue. After having recognized bone, the podosomes can organize themselves into a superstructure called the SZ, allowing for the polarized secretory phenotype of bone resorbing osteoclasts (Saltel et al., 2004; Luxenburg et al., 2007; Georgess et al., 2014). SZ formation is thus a prerequisite for bone resorption. The therefore required collagen removal from native bone surfaces has been ascribed to matrix metalloproteinases (MMPs) of bone lining cells and perhaps osteocytes (Chambers and Fuller, 1985; Nakamura et al., 2004; Abdelgawad et al., 2016; Delaisse et al., 2020).

### 3.2. A High Rate of Collagenolysis vs. Demineralization Is Necessary for Displacement of the SZ in the Resorption Cavity and Continuation of Resorption

**FIGURE 4:**
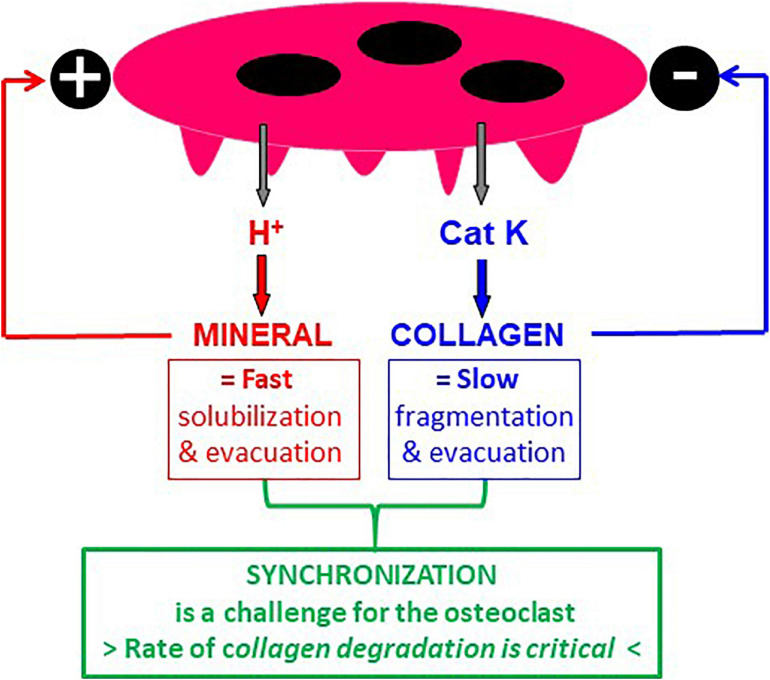
Mineral and collagen are not only substrates to be resorbed, but also regulators of osteoclastic bone resorption. It is well established that mineral is solubilized by protons and collagen by cathepsin K. However, it is often neglected (i) that mineral is an activator of resorption and collagen an inhibitor of resorption (see section 3), and (ii) that solubilization of mineral is by nature a faster process compared with collagen degradation and clearance. Hence, the challenge for the osteoclast is to degrade collagen at the same rate as demineralization – otherwise collagen accumulates and stops resorption.

The bone matrix consists of collagen embedded in mineral. Bone resorption thus requires demineralization, which is achieved by proton secretion (Baron et al., 1985), and collagen degradation, which is achieved by cathepsin K, a powerful proteinase degrading collagen once it is demineralized (Saftig et al., 1998; Garnero et al., 1999). Hence, one should be aware that presence of collagen can be “generated” in the excavation when the rate of demineralization exceeds the rate of collagen degradation. This collagen is then very likely to prevent SZ displacement in the excavation and to stop resorption just as mentioned in section 3.1 in relation with the initiation of a resorption event. So, collagen and mineral should not merely be considered as substrates to be resorbed, but also as important regulators of the resorptive activity: mineral activates resorption whereas collagen acts as a brake (Figure 4). Furthermore, solubilization of mineral is by nature a fast process, whereas collagen fragmentation and the evacuation of the collagen fragments is by nature more complex and slower (Figure 4). It is thus a challenge for the osteoclast to coordinate the speed of demineralization and collagenolysis, which by nature occur at very different rates (Figure 4). Collagen will automatically operate as a brake in case of excessive demineralization activity and will thereby protect bone against too high resorption. This brake is not activated when collagen degradation is as fast as demineralization, thereby allowing the SZ to move in the cavity.

**FIGURE 5:**
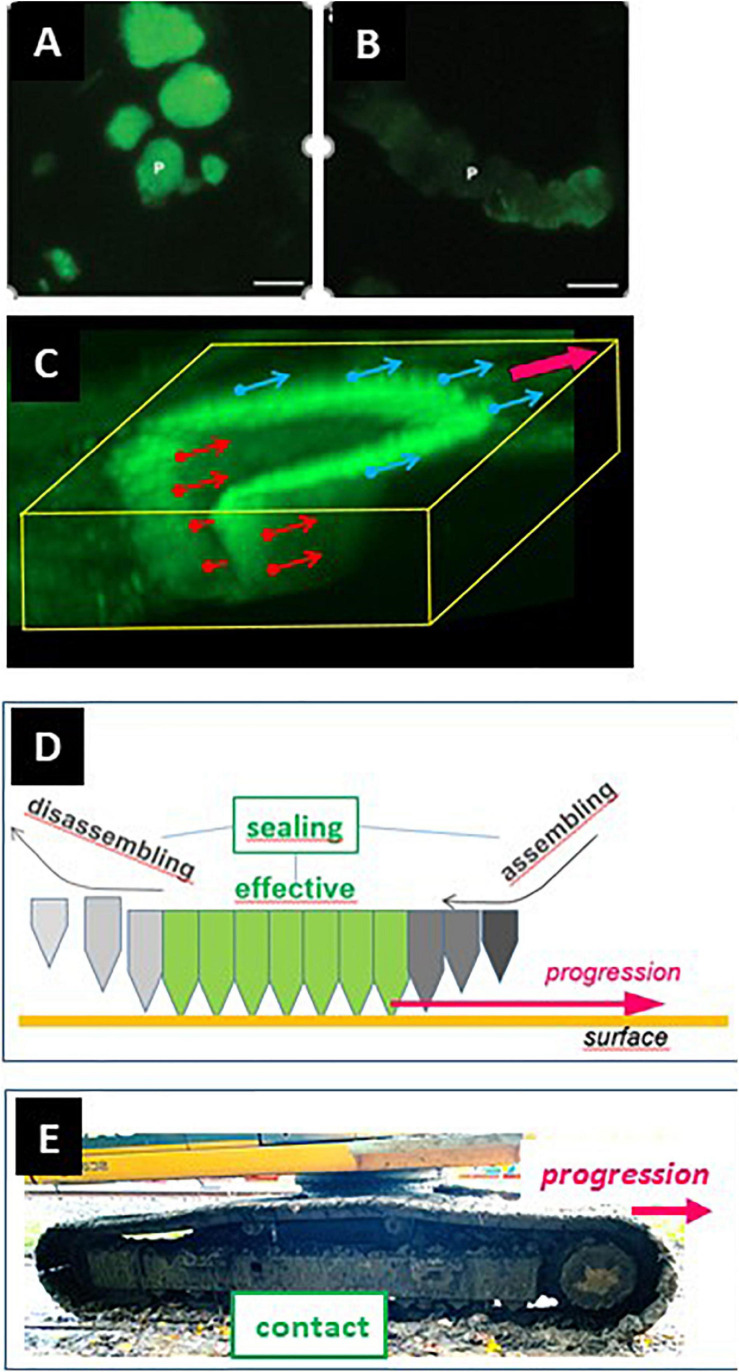
Absence of collagen on the cavity walls is a prerequisite for SZ displacement in a resorption cavity and for trench formation. **(A,B)** Bone slices were exposed to resorptive activity of human osteoclasts, stained for collagen immunoreactivity, and visualized through fluorescence microscopy [**panels (A,B)**, reproduced from Leung et al. (2011) with permission]. Pits **(A)** show strong signals (green). In contrast, trenches **(B)** show weak collagen signal (green). **(C)** Confocal picture showing the 3D configuration of the SZ of a trench forming osteoclast (unpublished). The SZ was visualized through staining of actin with phalloidin (Søe and Delaissé, 2017). The osteoclast is moving in the direction of the magenta arrow shown at the upper right corner. The small arrows originating from the SZ indicate the surface that must be devoid of collagen to allow the displacement of the SZ. The blue ones concern the displacement on the bone surface (and may depend on collagen degradation by MMPs of bone lining cells). The red ones concern the displacement along the cavity walls (and depend on collagen degradation by cathepsin K). **(D)** Displacement of the SZ is dependent on assembly of new podosomes at the leading edge and disassembly at the rear of the podosome band. Note that this means that the assembly site is at the outer rim of the SZ domain laying on the bone surface and of the inner rim of the SZ domain lining the cavity walls. Interestingly, these respective assembly sites correspond with the surfaces devoid of collagen shown in **panel (C)**. **(E)** Caterpillar track allowing simultaneous movement and contact. This picture is shown as a comparison with the posodosome band shown in **panel (E)**, to make understood that seal and movement do not exclude each other.

Evidence for this view is provided by the analysis of collagen remnants in the excavations – whether through immunoreactivity (Leung et al., 2011; Abdelgawad et al., 2014; Figures 5A,B), spectroscopy (Panwar et al., 2016), SEM (Søe and Delaissé, 2010; Panwar et al., 2016), or measurements of thickness of collagen fringes (Søe et al., 2013). Pits show almost always plenty of collagen remnants (Figure 5A) - meaning that collagenolysis has been slower than demineralization, thereby leading to accumulation of collagen which then does not allow the SZ to move in the pit and prevents transition to the trench mode. In contrast, trenches do not show presence of collagen (Figure 5B), which means that collagenolysis has been as fast as demineralization, and thus the rear part of the SZ could move along the walls of the cavity, which can therefore be continuously enlarged. Very thorough collagen removal along the side walls of trenches is in line with the highly polarized secretion of cathepsin K at the periphery of the ruffled border (Mulari et al., 2003; Lemaire et al., 2014) (see section 5). Besides, prolonged resorption is also seen if osteoclasts are seeded on pure mineral (Saltel et al., 2004; Geblinger et al., 2009; Wijenayaka et al., 2009), or on bone rendered anorganic (Søe et al., 2013) - which is consistent with the absence of the “collagen brake”.

The hypothesis that the collagenolysis/demineralization balance determines the ability to switch into the trench mode is further supported by pharmacological evidence (Søe et al., 2013). The presence of a low dose of carbonic anhydrase inhibitor slowing down demineralization vs. collagenolysis in osteoclast cultures on bone slices, leads to absence of collagen accumulation and a 2-fold increase in the proportion of trenches at the expense of pits (Søe et al., 2013). Thus, slowing down the demineralization vs. collagenolysis rate renders collagenolysis fast enough to prevent collagen accumulation, and so more trenches and less pits are generated. Conversely, the presence of a cathepsin K inhibitor slowing down collagenolysis, allows fast collagen accumulation, prevents formation of trenches, and concomitantly induces an increase in pit number (Søe et al., 2013; Panwar et al., 2016, 2017; Borggaard et al., 2020). Thus slowing down the collagenolysis vs. demineralization rate prompts a fast accumulation of collagen and thereby an early arrest of resorption, and so the transition to trench formation does not occur. These observations link collagen degradation rates vs. demineralization rates, transition from pit to trench resorption mode, SZ displacement, and prolongation of the resorption event. The association of these events stresses the contribution of collagen degradation for positioning the SZ and the ruffled border it surrounds (Figure 5C). Thereby the SZ appears as a navigation wheel operated by collagen degradation, and orienting the direction of resorption. (i) The displacement of the SZ along the freshly resorbed walls of the excavation is conditioned by fast degradation of demineralized collagen by cathepsin K (Figure 5C). Therefore, cathepsin K activity levels are expected to be especially critical for regulating the duration of resorption events/enlargement of resorption cavities (section 3.3). (ii) If dealing with native bone surfaces, one should also take into account the displacement of the leading edge of the SZ on the bone surface (Chambers and Fuller, 1985; Everts et al., 2002; Delaisse et al., 2020). Collagen degradation on the bone surface immediately in front of the moving osteoclast, is conditioned by removal of collagen by MMPs of the bone lining cells known to support the osteoclastic resorptive activity (Chambers and Fuller, 1985; Nakamura et al., 2004; Abdelgawad et al., 2016; Delaisse et al., 2020; Figure 5C).

### 3.3. Clinical Significance of High Levels of Active Cathepsin K for Enlargement of Resorption Cavities

The need for sufficient cathepsin K to allow transition from pit to trench mode is clearly demonstrated by the concomitant abolishment of trench formation and increase in pit formation in response to cathepsin K inhibitors (Søe et al., 2013; Panwar et al., 2016, 2017; Borggaard et al., 2020), as mentioned in section 3.2. It is interesting to note the relevance of this finding to the variations in osteoclast aggressiveness in the human population. Healthy blood donors show a great inter-individual variation in cathepsin K expression levels in their osteoclasts (Merrild et al., 2015), and the levels of active cathepsin K (directly titrated in the osteoclasts from these respective donors) explain 70% of the variations in prevalence of trench formation by the osteoclasts generated from these different donors (Borggaard et al., 2020). Furthermore, the age of the donor proves to be an important determinant of levels of both active cathepsin K and prevalence of trench formation (not of pit formation) (Møller et al., 2020c), thus suggesting that enlargement of resorption cavities is an important contributor of the age effect on bone quality. Menopause status is associated with increased levels of active cathepsin K (Møller et al., 2020c) whereas estrogen represses cathepsin K levels (Mano et al., 1996) and leads to more collagen remnants in the excavations in osteoclast cultures (Parikka et al., 2001). Osteoclasts generated from males were reported both to express higher cathepsin K levels and to generate more trenches (Merrild et al., 2015). Glucocorticoids were reported to increase both cathepsin K levels (Swanson et al., 2006) and trench formation (Søe and Delaissé, 2010). Interestingly, the number of nuclei per osteoclast (supporting protein-synthesis power) is another characteristic that shows variability amongst donors (Møller et al., 2020b) and that positively correlates with cathepsin K levels (Møller et al., 2020a) and prevalence of trenches (Møller et al., 2020c). Overall, one may conclude that the differences in active cathepsin K levels amongst individuals will affect the duration of the resorption events and the size of the resorption cavities. Whether this mechanism also contributes to differences in bone resorption depending on inflammatory conditions (Redlich and Smolen, 2012; Madel et al., 2020) or, the skeletal site (Everts et al., 1999) remains to be investigated. Still, regarding the latter, it is of interest to mention that osteoclasts generated from human bone marrow show a higher propensity to make trenches, compared with those generated from blood (Merrild et al., 2015), and that mouse long bone osteoclasts show higher levels of cathepsin K than calvaria osteoclasts (Everts et al., 1999).

## 4. Simultaneous Resorption and Osteoclast Displacement in the Trench Resorption Mode

According to the classical pit model, the osteoclastic resorptive activity is stationary and cannot occur simultaneously with osteoclast displacement (Lakkakorpi and Vaananen, 1991; Saltel et al., 2004; Georgess et al., 2014). The direct demonstration of the trench resorption mode through time lapse obliges to reconsider this classical view (Søe and Delaissé, 2017). Sections 2 and 3 focused on the displacement of the SZ and the resorption compartment it delineates. They highlighted that a high rate of collagenolysis vs. demineralization is permissive for this displacement. The present section reports additional observations throwing light on SZ displacement. Furthermore, it takes into consideration the displacement of the cell body, which brings the resorption compartment to critical areas of the bone surface to be resorbed.

### 4.1. SZ Displacement

As mentioned above, the SZ is known to consist of a superstructure of densely interconnected podosomes (Saltel et al., 2004; Luxenburg et al., 2007; van den Dries et al., 2019). Interestingly with respect to SZ displacement, podosomes have a half-life of about 2-12 min (Linder, 2007; Luxenburg et al., 2012). The SZ is thus undergoing constant remodeling. This is very well reflected by the somewhat unsteady appearance of the SZ of pit forming osteoclasts observed in time-lapse (Søe and Delaissé, 2017), and in accordance with the concept of a “dynamic” SZ (Stenbeck and Horton, 2000; Saltel et al., 2004; Geblinger et al., 2010). Therefore, one may envision that in trench mode, the SZ remodeling is organized in such a way that new podosomes are appended at the leading edge of the podosome band and removed at the rear edge of the podosome band (Figure 5D). It is remarkable that these podosome appositional areas of the SZ exactly correspond to the areas where full clearance of collagen is critical for SZ displacement: i.e., to the outer rim of the SZ domain positioned on top of the bone surface, and the inner rim of the SZ domain positioned inside the excavation (Figure 5C) (see section 3). Of note, movement of “podosome groups” in migrating cells (Linder and Kopp, 2005) as well as of podosome belts in osteoclasts (Tehrani et al., 2006) is also achieved by assembly at the front and disassembly at the rear. Together these observations lead to propose that the coordinated formation and removal of podosomes allows a unidirectional displacement of the SZ, while an effective seal is maintained in the center of the podosome band. This working principle is reminiscent of a caterpillar track simultaneously allowing movement and strong contact (Figure 5E). The mechanism coordinating site specific podosome recruitment and migratory cues of the cell body remains to be investigated.

### 4.2. Cell Body Displacement

While SZ displacement responds to “rigidity-sensing” (van den Dries et al., 2019), and therefore to collagen degradation (section 3), the cell body responds to signals ordering where erosion should occur (Niwa et al., 2013; Brylka and Schinke, 2019), and guides the resorption compartment over the bone surfaces to be eroded. Time lapse and confocal microscopy show that osteoclasts forming a trench have similar migratory features as purely migrating osteoclasts (Wheal et al., 2014): (i) lamellipodia ahead of the front part of the SZ, and (ii) a rear edge showing intermittent and abrupt displacements compared with the steady speed of the SZ and the ruffled border, as can be very well appreciated on kymographs (Søe and Delaissé, 2017). This intermittent displacement of the rear results in alternations of stretching and retraction of the cell body (Wheal et al., 2014; Søe and Delaissé, 2017). In purely migrating osteoclasts, stretching was ascribed to attachment of the rear and proposed to relate to accumulation of α_*v*_β_3_ integrins as seen in neutrophils, whereas retraction was shown to result from disassembly of cell adhesion complexes at the rear (Wheal et al., 2014). In this respect, it is of interest that trench-forming osteoclasts show abundant α_*v*_β_3_ integrins at the rear (Mulari et al., 2003), allowing them to attach in the same way.

A recent observation shows particularly well that detachment of the rear is indeed a critical event involved in the displacement of trench-forming osteoclasts: this detachment is prevented by a low dose of chloroquine, a compound that interferes with lysosomal function and trafficking (Borggaard et al., 2020). This low dose does not affect pit formation, nor the transition from pit to trench, nor displacement of the front, nor resorption speed (Borggaard et al., 2020). The immobilization of the rear makes the osteoclast stretching extensively, but at some point, the front of the osteoclast detaches and bounces backward while the rear remains attached (Borggaard et al., 2020). This reflects a progressive imbalance in the strength of cell adhesion between the front and the rear. Furthermore, this imbalance coincides with prevention of exocytosis of resorption products, which then accumulate at the rear (Borggaard et al., 2020) (see section 5). This dual effect of chloroquine thus points to a mechanism that connects exocytosis with coordination of attachment/detachment of the rear and the front of trench forming osteoclasts (Borggaard et al., 2020) (see section 5). In line with this, resorption products were proposed to disrupt cell adhesion complexes upon termination of bone resorption (Wilson et al., 2009). Again, this mechanism remains to be investigated.

## 5. Evacuation of the Resorption Products

Maintenance of contact of the SZ with fully mineralized collagen, and hence long duration resorption events, does not only demand efficient collagen degradation, but also fast evacuation of the collagen fragments from the resorption compartment (Nesbitt and Horton, 1997; Salo et al., 1997). As mentioned in section 3.2, proper resorption requires thorough fragmentation of demineralized collagen in the resorption compartment, and at the electron microscopy level, it appears to melt between the folds of the ruffled border (Miller, 1977; Stenbeck and Horton, 2000). Accordingly, the evacuation of these fragments is proposed to involve fluid phase and clathrin-mediated endocytosis (Mulari et al., 2003; Stenbeck and Horton, 2004). The internalized vesicles are then crossing the cytoplasm and are exocytosed at the basolateral domain opposite to the ruffled border which is called the functional secretory domain (FSD) (Salo et al., 1996, 1997; Coxon and Taylor, 2008; Hirvonen et al., 2013; Ng et al., 2019). The half-life of the endocytosed material is only 22 min (Stenbeck and Horton, 2004). Striking pictures of release of resorption products at the FSD were reported (Nesbitt and Horton, 1997; Salo et al., 1997; Rogers et al., 2011; Ng et al., 2019), but the mechanism of this exocytosis is poorly known. The coordination of endocytosis at the RB, transcytosis, and exocytosis at the FSD is suspected to be important to cycle integrins and plasma membrane, and considered necessary to meet the demands of the ruffled border for large amounts of plasma membrane (Palokangas et al., 1997; Stenbeck, 2002; Mulari et al., 2008). Overexpression of dynamin - which pinches off the vesicles budding inside the cytoplasm (von Kleist and Haucke, 2012) - makes osteoclasts generating long trenches instead of round pits in bone slices (Bruzzaniti et al., 2005). This effect points to endocytosis as a critical component of the mechanism allowing trench formation/sustained resorption. We mention here two characteristics of this evacuation that appear different in the trench mode compared to the pit mode. First, the spatial organization of this evacuation system is distinct, as based on the positioning of specific markers, such as clathrin, dynamin and α_*v*_β_3_ for evacuation, compared to Rab7 and v-H^+^-ATPase for secretion (Mulari et al., 2003). The ruffled border of pit forming osteoclasts appears organized in two concentric zones: internalization of resorption products occurs in the middle part, whereas the resorption agents are secreted at its periphery (Mulari et al., 2003). In trench forming osteoclasts, these two zone appear as imbricated crescents. Internalization occurs at the inner crescent whereas the resorption agents are secreted at the level of the outer one (Mulari et al., 2003) (thereby favoring collagen removal along the cavity walls as mentioned in section 3.2). Furthermore, the FSD is at the opposite side of the ruffled border, which is thus distal to the bone surface in the pit mode, but at proximity to the bone surface in the trench mode (Stenbeck and Horton, 2004), thereby allowing exocytosis at the FSD to interfere with cell attachment (Borggaard et al., 2020). With respect to the latter, a second point of interest was highlighted by time-lapse: trench forming osteoclasts show evidence for traffic of big vacuoles carrying resorption products from the front to the rear, where they disappear (Søe and Delaissé, 2017; Borggaard et al., 2020). As mentioned in section 4, chloroquine induces accumulation of these vacuoles at the rear, indicating impaired exocytosis (likely because digestion of their content is prevented by the presence of chloroquine) (Borggaard et al., 2020). This evacuation route is poorly characterized, but is intriguing because it is not detected in the pit mode, and is blocked simultaneously to the prevention of detachment of the rear/attachment of the leading edge (Borggaard et al., 2020) (see section 4). These observations highlight a link between a seemingly trench-specific membrane cycling route, and displacement of osteoclasts (see section 6). This link deserves attention in future studies.

## 6. Evidence for Pre-Cavitation Activities Preparing Resorption

**FIGURE 6:**
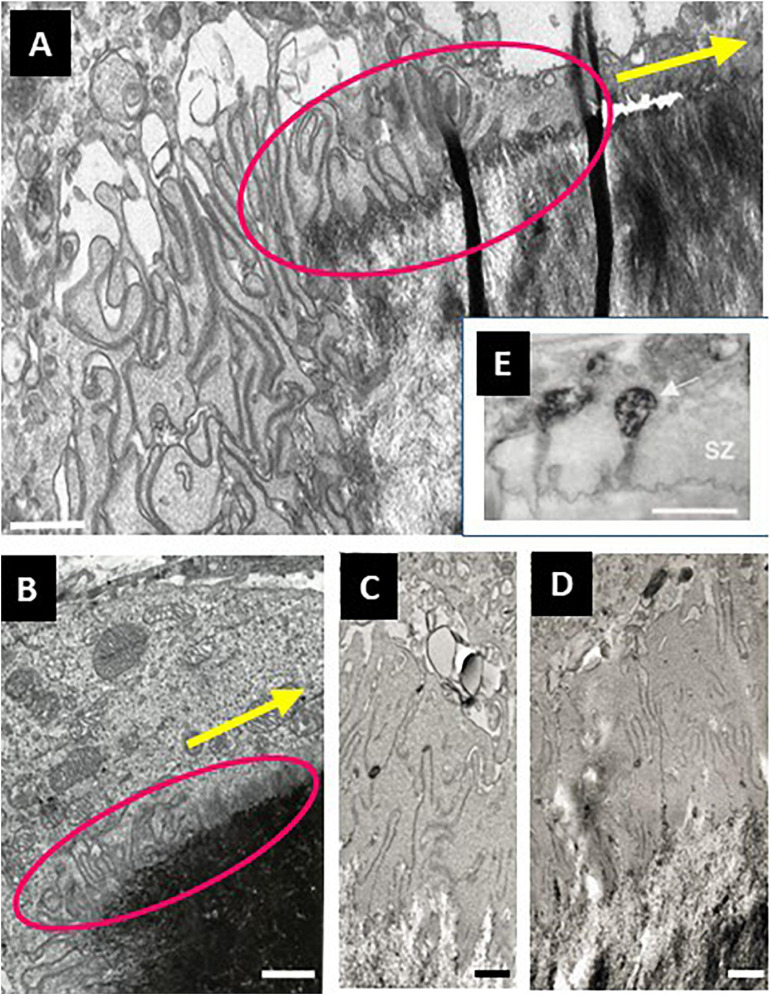
Electron microscopy pictures in the area of pre-cavitationsurfaces of trench forming osteoclasts: peculiarities of the CZ, nascent ruffled border, and underlying bone surface. **(A–D)** Electron microscopy pictures of osteoclasts sectioned parallel to their resorption/migration axis were obtained in the same way as in Figure 1B (unpublished, A is a higher magnification of the pre-cavitation area shown in Figure 1B). **(A,B)** Pre-cavitation areas are framed in magenta and the resorption/migration axis is shown by the yellow arrow. Note the gradient of plasma membrane folds over the pre-cavitation surface toward the edge of the cavity, and as well the corresponding gradient of alterations of the surface of the bone matrix. Interestingly, these folds appear in a “clear cytoplasm” typical of the CZ/SZ. **(C–E)** Narrow canals are visible across the CZ suggesting communication between the cytoplasm and the pre-cavitation surface. It is speculated that these canals may represent a preparative step of ruffled border formation. Interestingly, the canals of the CZ of a trench forming osteoclast in E are connected to vacuoles at the cytoplasmic side and show clear openings toward the bone surface [reproduced from Mulari et al. (2003) with permission). Scale bars 1 μm.

**FIGURE 7:**
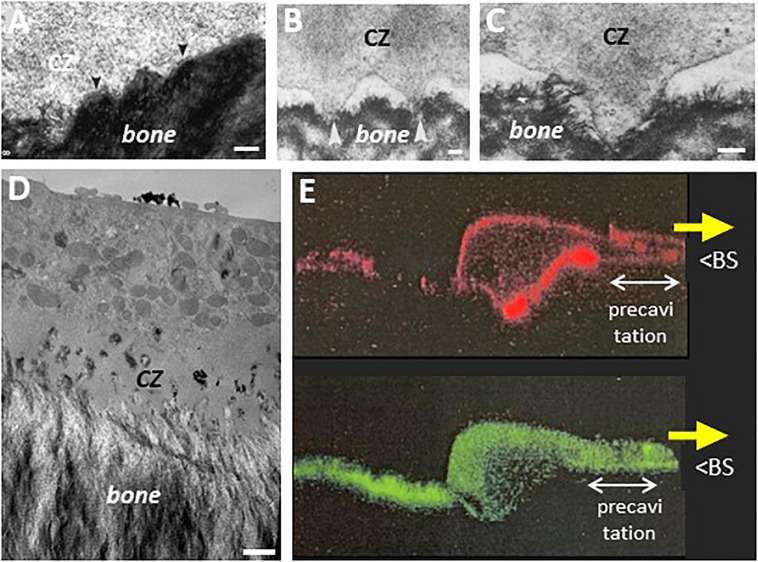
Electron microscopy pictures in the area of pre-cavitation surfaces: podosomes, superficial disruptions of the bone matrix, and evidence for uptake of resorption products. **(A)** Uneven interface between CZ and underlying bone surface that received attention in Pierce (1989) [rat maxillary osteoclast, reproduced from Pierce (1989)]. **(B,C)** Podosomes in the CZ of hen osteoclast. Note they protrude in the matrix of medullary bone [reproduced from Zambonin-Zallone et al. (1988) with permission]. **(D)** EM picture of the pre-cavitation area of an osteoclast sectioned along the resorption/migration axis (obtained as in Figures 1B, 6, unpublished). Note the disrupted bone surface, the nascent ruffled border in the CZ, and the electron dense vacuoles in the CZ, similar to the phagocytic features reported earlier in this area (Bonucci, 1974; Pierce, 1989). **(E)** Confocal picture of a trench forming osteoclast sectioned along its resorption/migration axis and perpendicularly to the bone surface (unpublished). The osteoclast is moving in the direction of the yellow arrow. Human osteoclasts were obtained and analyzed on dentine as in Nesbitt and Horton (1997) (red: actin; green: collagen fragments stained with a collagen antibody as in Nesbitt and Horton (1997); unpublished picture of S. Nesbitt (The Royal Institution, London) with kind permission). BS: bone surface. Note the ruffled border and the SZ are at the typical position seen in trench forming osteoclasts. Note the intracellular collagen signal in the pre-cavitation area and all along the basolateral domain up to the rear of the osteoclast (- which fits the route of resorption products shown by time lapse). Scale bars in **(A)** 100 nm; **(B)** 1 μm; **(C)** 250 nm; **(D)** 1 μm.

**FIGURE 8:**
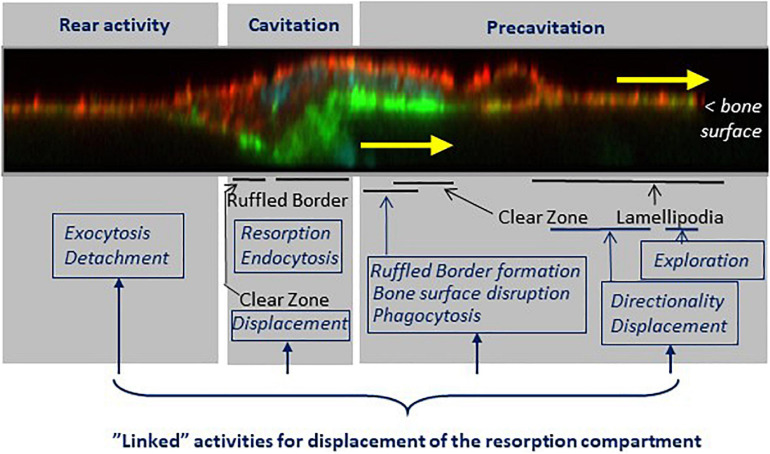
The shift from the pit to the trench resorption mode goes along with a specific arrangement of cellular domains responsible of the respective tasks supporting trench formation. Key features (black fonts) and related tasks (blue fonts) are mapped on a confocal picture of a trench forming osteoclast sectioned along its resorption/migration axis and perpendicularly to the bone surface (actin stained with phalloidin, green; α_*v*_β_3_ stained with α_*v*_β_3_ antibody, red, unpublished). The osteoclast is moving in the direction of the yellow arrows. (i) The pre-cavitation domain harbors lamellipodia and the front part of the SZ. This domain plays a major role in exploration, displacement and in determining the direction of resorption. It supports ruffled border formation, damages superficially the bone surfaces, and shows phagocytic events. (ii) The cavitation domain shows the same well known characteristics as in the pit mode: a fully developed ruffled border, thorough resorption and endocytosis. A critical difference, however, is that the cavitation harbors part of the SZ, which moves along the cavity walls. (iii) The rear appears to be an exit site for resorption products, and their release is involved in the mechanism allowing the rear to detach from the bone surface, thereby allowing the cell body to migrate. Of note, the continuous displacement of the resorption compartment requires the coordination of all these tasks. See sections 4 to 6.

An interesting implication of the trench resorption mode is that the leading edge of the cell body is laying on the bone surface to be eroded. This can be clearly appreciated when sectioning trench-making osteoclasts parallel to their resorption/migration axis, and observing them through confocal and electron microscopy. These images show lamellipodia at the very front, followed by an actin rich cytoplasm [called clear zone (CZ) in electron microscopy terminology and appearing to coincide with the SZ defined in confocal microscopy], and only thereafter the actual cavitation together with the ruffled border (Figures 1B, 6A,B, 7E, 8; Møller et al., 2018). Interestingly, nascent folds of ruffled border are visible over this pre-cavitation surface. This means that the “import” of membrane into the ruffled border [as a compensation for ruffled border uptake at the resorption cavity (Palokangas et al., 1997; Stenbeck, 2002; Mulari et al., 2008)], is directed to the leading edge of trench forming osteoclasts. This targeted import supports the displacement of the ruffled border and is part of the mechanism displacing the resorption compartment. Morphologically, this ruffled border formation appears as an increasing number of tiny folds of the plasma membrane with deeper and deeper interdigitations (Figures 6A,B). This gradient is immediately adjacent to an area of the CZ crossed by tiny canals (Figures 6C-E), some of which open extracellularly (Figure 6E). The continuum between these features suggests that the recruitment of these tiny canals and their fusion with the plasma membrane could represent the first step of the ruffled border formation mechanism. Such a link between CZ and ruffled border generation would explain the intriguing inverse variation in CZ size and ruffled border size, in response to variations in small GTPases or PLEKHM1 (Reinholt et al., 1999; Zhao et al., 2001; Alakangas et al., 2002; Van Wesenbeeck et al., 2007). Explorations of these pre-cavitation areas may refine the knowledge on ruffled border formation, which is often merely claimed to be due to massive fusion of lysosomes with the plasma membrane (Ng et al., 2019).

Furthermore, the electron microscopy pictures of these osteoclast sections reveal superficial disruptions of the matrix beneath the CZ (Figures 6A, 7A-D). The attention on an irregular nanotopography beneath the CZ has already been drawn in earlier publications (Holtrop and King, 1977; Zambonin-Zallone et al., 1988; Pierce, 1989; Sasaki et al., 1989), but their significance could not be understood, as the pit model did not allow thinking of them as bone surfaces to be resorbed. It is tempting to reconsider these alterations as part of the trench resorption process where they would represent the initial step of matrix disruption. One may speculate that the above mentioned canals crossing the CZ deliver resorption agents onto these pre-cavitation surfaces (Figure 6), and the fact that some of these canals are connected to vacuoles is compatible with this speculation (Figure 6E). Superficial disruptions may also result from podosomes (Figures 7B,C), which are associated with proteinases (Linder, 2007; Saltel et al., 2008), and exert dislocating mechanical forces by penetrating into the matrix (van den Dries et al., 2019). A link between podosomes, SZ/CZ, and nanotopography has been mentioned (Holtrop and King, 1977; Zambonin-Zallone et al., 1988; Shemesh et al., 2016). It is not unlikely that these superficial disruptions would lead to release of matrix clumps (including mineral), which would then be taken up by phagocytosis due to their size. This is very much in contrast with the mode of uptake of the thoroughly solubilized matrix “melting” between the deep folds of the fully developed ruffled border (see section 5). This hypothesis of phagocytosis in the pre-cavitation area is supported by electron dense vacuoles in the pre-cavitation area of trench forming osteoclasts sectioned along their migration/resorption axis (Figure 7D). Intracytoplasmic vacuoles of the same appearance were reported in the earlier literature at the level of the CZ (Pierce, 1989) and of poorly developed ruffled border (Bonucci, 1974). Evidence for uptake of collagen at that level is provided by collagen immunoreactivity in the osteoclast cytoplasm covering the pre-cavitation surface (Figure 7E). All these observations taken together suggest that the superficial disruptions on the pre-cavitation surfaces are directly due to osteoclasts and do not merely represent pre-existing irregularities of the bone surface. Regarding the electron microscopy phagocytic features, one should also mention that they may well relate to the vacuoles identified in time lapse of trench making osteoclasts (section 5). (i) They transport resorption products from the leading edge to the rear where they disappear. (ii) Their site of origin at the front edge of the cavity would explain their detection specifically in the trench mode. (iii) Phagocytosis would explain the large size of these vacuoles. (iv) This phagocytic process requires membrane consumption at the front, which may put at risk attachment/protrusion ability of the front, unless membrane is returned to the plasma membrane by exocytosis at the rear. Blockage of exocytosis at the rear by chloroquine (section 5) would prevent this return and therefore lead to detachment of the front (section 4).

Thus, the trench resorption mode allows for a series of pre-cavitation activities that may contribute importantly to osteoclast aggressiveness, but that are not taken into account in the standard models of osteoclastic bone resorption.

## 7. Key Messages and Perspectives

**FIGURE 9:**
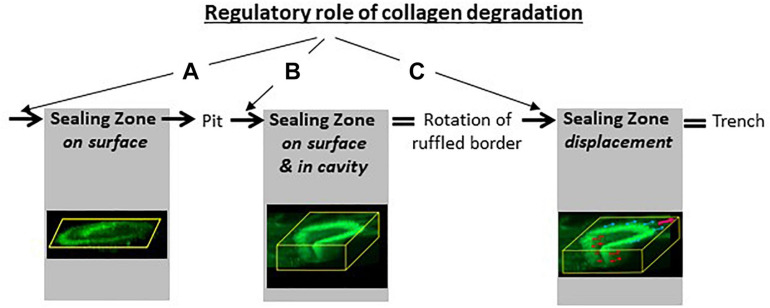
Collagen degradation as a “regulator” of SZ and bone resorption. Next to its obvious direct role in the formation of a cavity, collagen degradation has a key role for SZ formation **(A)**, orientation **(B)** and displacement **(C)** (see section 3). **(A)** The formation of a planar SZ on the bone surface depends on thorough collagen degradation by MMPs of bone lining cells and orients the ruffled border against the bone surface for drilling a pit (see Figure 1A). **(B)** The movement of part of the SZ into the pit depends on thorough collagen removal from the pit by cathepsin K and orients the ruffled border against the cavity wall to resorb parallel to the bone surface (see Figure 2B). **(C)** The continuous displacement of the SZ parallel to the bone surface depends on thorough collagen removal from the cavity walls by cathepsin K and from the bone surface by MMPs of bone lining cells, and leads to the formation of a trench (sections 3.2 and 4). The confocal pictures are from Figures 1 and 5.

• The observations reviewed herein lead to envision the mechanism that allows the osteoclast extensively enlarging its resorption cavity by making a trench. As explained in section 1, this enlargement is at the basis of bone erosion and pathological bone destruction, and therefore its mechanism is important to know.
• This mechanism corresponds to a unique osteoclastic phenotype characterized by integrated resorption and migration activities. Figure 8 shows a spatial model of this phenotype, taking into account the characteristic configuration of a trench-forming osteoclast: its front on the bone surface and its main body in the resorption cavity. (i) The front domain laying over the bone surface is responsible for guiding resorption on the bone surface (see section 4), for continuously generating new ruffled border (which appears to arise from inside the CZ itself) (see section 6), and for disrupting superficially the bone surface and taking up matrix clumps through phagocytosis (see section 6). (ii) The “cavity domain” corresponds to the classical resorption compartment. Importantly, however, this is also where the SZ surrounding the ruffled border has to displace itself along the cavity wall (see sections 3 and 4). (iii) The “rear domain” is responsible for a process linking the release of resorption products and the disassembly of attachment complexes between the cell and he bone surface (see section 5). The standard model of the resorption mechanism shown in Figure 1A thus gives only a partial view of the machinery involved in the “large size” resorption events.
• The special role of the SZ has to be underscored. Rather than anchoring the bone resorption compartment, it emerges as the structure transporting the resorption compartment/ruffled border (just like the structure bearing the boring head in a tunneling machine) (see sections 2 and 4), and it is closely associated with ruffled border formation at the leading edge (see section 6). Accordingly, the SZ governs the “resorption route” during the long duration resorption events (Figure 9): as explained in section 2, it directs the resorptive activity first against the bone surface and thereafter against the cavity wall. This resorption route is in line with the fact that 90% of trench resorption events start as a pit (Søe and Delaissé, 2017).

• The principle linking collagen removal, SZ formation and “initiation” of resorption is well known (see section 3.1). Here we highlight that this principle also rules all the subsequent steps involved in the “enlargement” of a resorption cavity, since collagen removal must be as fast as demineralization to allow SZ displacement in the cavity, thereby allowing trench formation (see section 3.2) (Figure 9). Accordingly, it is of big interest to investigate which are the upstream effectors that affect the coordination between collagen degradation and demineralization, as already discussed by Søe et al. (2013).
• The collagen degradation required for SZ displacement in the excavation is performed by cathepsin K. This stresses that in addition to the obvious role of cathepsin K to resorb the bulk of collagen of the bone matrix, thereby creating a cavity, cathepsin K has also a regulatory role to remove the “negative collagen signaling” toward osteoclasts. This regulatory role is permissive to initiate and continue cavity enlargement. Therefore, high levels of the collagen degrading cathepsin K is at the heart of the resorption efficacy of trench forming osteoclasts (Figure 9).
• This observation draws the attention on cathepsin K as a favorite target for anti-resorptives in the clinic (especially in cases of aging, glucocorticoid treatment, and menopause: see section 3.3). The upcoming cathepsin K exosite inhibitors appear interesting candidates, since they inhibit specifically collagen degradation without inhibiting the other cathepsin K activities, and therefore avoid the side effects of odanacatib (Panwar et al., 2016, 2017). Also from a diagnostic point of view, the variation in cathepsin K activity levels amongst individuals may be interesting to consider, when evaluating the risk of bone fragilization (see section 3.3).
• Understanding extended bone erosion requires thinking of osteoclasts beyond the standard bone resorption models, and therefore paying attention in the coordination between demineralization, collagen degradation, podosome dynamics in the SZ, continuous ruffled border formation and secretion of resorption agents at the leading edge, release of resorption products at the rear, and cell body displacement.

## Data Availability Statement

The original contributions presented in the study are included in the article, further inquiries can be directed to the corresponding author.

## Author Contributions

J-MD and KS generated the concept. TA, NM, and AR provided the unpublished transmission electron microscopy pictures. J-MD wrote the manuscript. All authors approved the final text.

## Conflict of Interest

The authors declare that the research was conducted in the absence of any commercial or financial relationships that could be construed as a potential conflict of interest.
